# P-564. Long Term Follow Up of Rapid or Same-day Start of Antiretroviral Therapy (ART) in Patients Living With HIV (PWH) in the Midwestern US

**DOI:** 10.1093/ofid/ofae631.762

**Published:** 2025-01-29

**Authors:** Joan Duggan, Eric G Sahloff

**Affiliations:** University of Toledo Medical Center, Toledo, Ohio; Univ of Toledo, Toledo, OH

## Abstract

**Background:**

Current DHHS guidelines recommend starting ART in PWH immediately or as soon as possible after HIV diagnosis to improve ART uptake, linkages to care and virologic suppression (VS). But there is little data measuring the longitudinal impact of rapidly starting ART. We previously showed that rapidly starting ART significantly decreased time to VS. This study compares the longitudinal effects of rapid ART start versus delayed ART initiation on retention in care and VS in a previously defined cohort of PWH.

**Figure:**
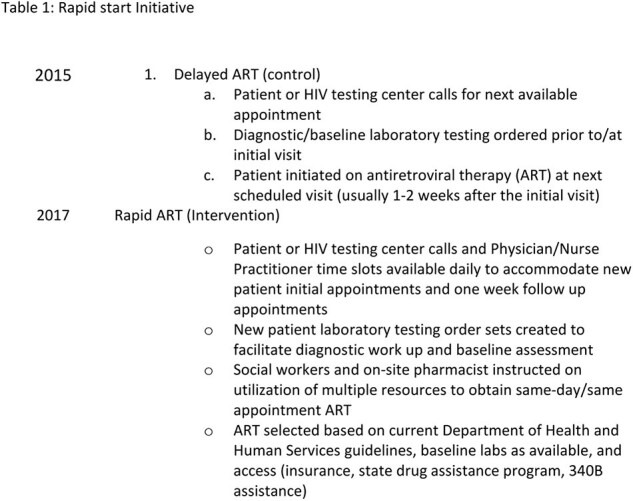

**Methods:**

IRB-approved follow up study of a single-center, retrospective cohort. The original cohort included all newly diagnosed, antiretroviral-naïve adult patients seen in the Ryan White Clinic (RWC) at a single site for their initial visit between 1/1/15-12/31/15 (delayed ART) and 1/1/17-12/31/17 (rapid ART): Table 1 and Table 2. In the original cohort, primary outcome of time from HIV diagnosis to VS [VL < 200 copies/ml (cp/ml)] was compared between groups. For the current study, the primary outcomes of VS (VL < 50 cp/ml) and retention in care (documented visit at study site or documented transition of care) were measured in the original cohort participants from 1/1/23-12/31/23. Reasons for lack of retention in care were death and loss to follow up.

**Figure:**
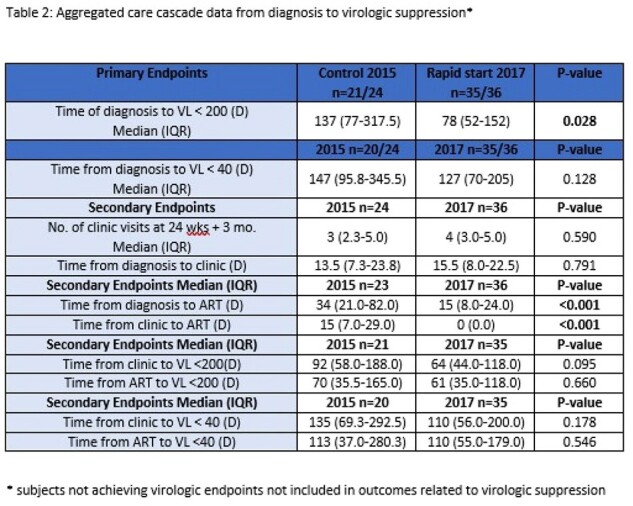

**Results:**

In the original cohort, 72 patients were screened and 60 met inclusion criteria; (24 delayed ART, 36 rapid ART). The long-term outcomes for VS and retention in care are shown in Table 3. 11/24 (delayed) and 23/36 (rapid) remained engaged in care at the study institution (p=.193). 9/11 (82%) and 22/23 (96%) maintained VS (p=.239), in the delayed ART and rapid ART, respectively. Participants were more likely to be retained in care (at study site or with documented transition in care) in the rapid ART group (11/22 delayed ART vs. 31/35 rapid ART, p=.002) If not retained in care at the study site, delayed ART participants were more likely to be lost to follow up versus having a documented transition in care or death [11/13 delayed ART vs 4/13 rapid ART, (p=.015)].

**Figure:**
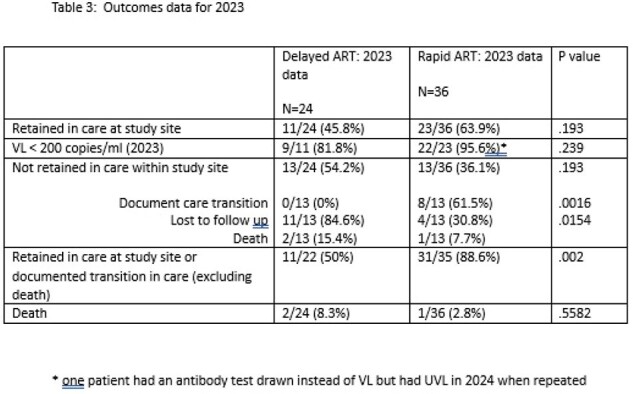

**Conclusion:**

A previous study showed a shortened time to VS with rapid start of ART. Long-term retention in care may be significantly improved with rapid start of ART and requires further study. Rapid start may also demonstrate a trend towards increased VS.

**Disclosures:**

**All Authors**: No reported disclosures

